# Changing ophthalmic practice during the COVID-19 pandemic in Uganda

**Published:** 2020-09-01

**Authors:** Raheel Rizwan Kanji, Simon Arunga

**Affiliations:** 1 Ophthalmology resident: Mbarara University of Science & Technology, Mbarara, Uganda, Kenya.; 2 Ophthalmologist: Mbarara University of Science & Technology, Mbarara, Uganda, Kenya.

**Ophthalmologists in Mbarara, Uganda explain how they have adapted their eye services during the pandemic.**

**Figure:**
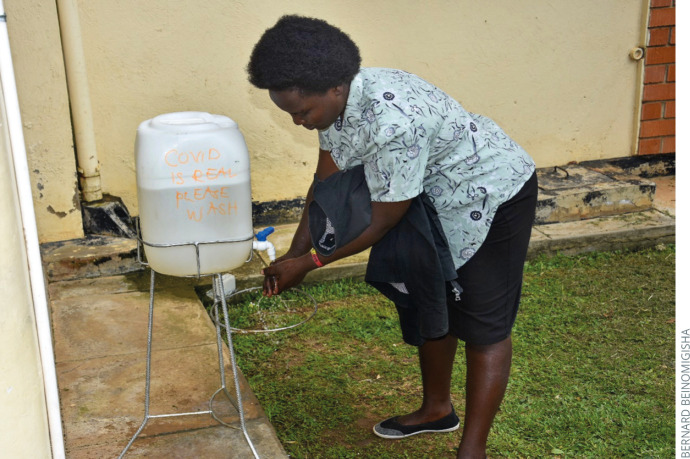
A patient washes hands before accessing the clinic building at MURHEC. **UGANDA**

Mbarara University and Referral Hospital Eye Centre (MURHEC) is a government-owned tertiary referral eye unit that provides eye care to the South Western part of Uganda, providing over 10,000 consultations per year. It also offers residency training in ophthalmology and currently has 20 residents.

## Patient care

Patient care services have been scaled down. We have had to turn away many patients (except emergencies and priority cases) and give longer appointments for chronic care cases. All elective surgery has been cancelled. This has seen a drop in consultations from 100 patients per day to less than 20.

We introduced a triage desk and a checklist which helps to assess which patient can be seen. The triage checklist is administered verbally and covers the following areas:

- Any history of fever, flu-like symptoms, cough or any upper respiratory symptom (these patients are immediately sent to the emergency department)
- Presenting complaint: patients with a history of acute loss of vision, sensitivity to light, eye pain, trauma, and severe acute discharge are considered as emergency cases and allowed to see a clinician.

**Figure:**
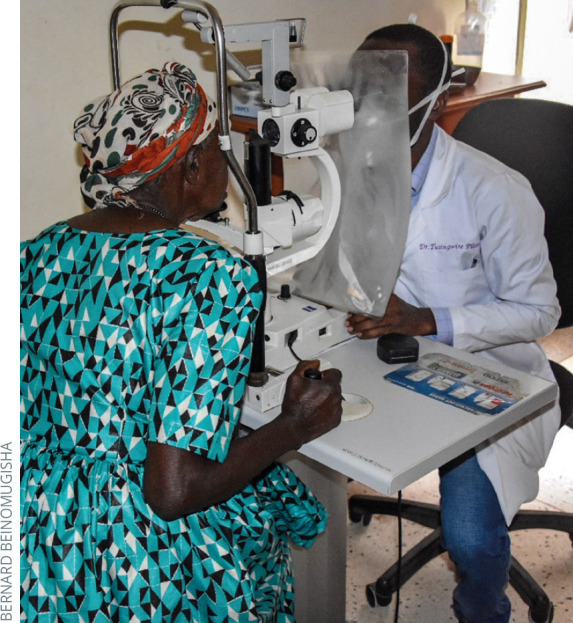
A clinician behind a protective shield examines a patient on the slit lamp. **UGANDA**

We introduced mandatory hand washing facilities outside the main entrance and require everyone entering the building (including staff members) to wash their hands with soap and water before they enter.

In addition, we introduced alcohol hand sanitiser dispenser stations at different points within the building.

## Staffing

Staffing has been reduced by three-quarters: from 32 full-time staff members to a rota of 8, consisting of a supervising head nurse, a cleaner, a receptionist, an optometrist, an ophthalmic nurse (who also helps in administering anaesthesia and surgical scrub in theatre), a clinical officer and an on-call resident under the supervision of a specialist/consultant.

We have put several measures in place to maximise staff sensitisation and protection:

- Staff received training on COVID-19 and infection control
- The Ministry of Health and the Uganda Medical Association have shared information and resources. Other sources of information include the online Zoom conference hosted by the International Centre for Eye Health (ICEH) and Royal College of Ophthalmologists recently: [**https://www.cehjournal.org/news/covid-19-and-ophthalmology-in-african-eye-units-conference-recordings-now-available/**]
- Personnel have been provided with face masks (disposable) and disposable gloves.
- Protective shields (locally made) have been added to the slit lamps as breath guards, to minimise close contact between the ophthalmologist and patients.
- Clinicians were encouraged to minimise procedures that may require close contact with patients, such as direct ophthalmoscopy.

## Residency training

Following a directive by Uganda’s president to close all schools, residency training was halted. The residents were sent into recess to use this time to work on their master’s research protocols. The more senior residents are available to support clinic work.

## Research activities

All ongoing clinical research that is not related to COVID-19 has been paused following a directive from the Uganda National Council of Science and Technology.

